# Collision Integrals for Transport in Plasmas: The Phenomenological Approach

**DOI:** 10.3390/e28030325

**Published:** 2026-03-13

**Authors:** Fernando Pirani, Massimiliano Bartolomei, Gianpiero Colonna, Annarita Laricchiuta

**Affiliations:** 1 Dipartimento di Chimica, Biologia e Biotecnologie, Università di Perugia, 06123 Perugia, Italy; 2 Instituto de Física Fundamental—CSIC, 28006 Madrid, Spain; 3 CNR ISTP Bari Section, 70126 Bari, Italy

**Keywords:** phenomenological approach, collision integrals, transport properties in plasmas, ablated species

## Abstract

The accuracy of transport properties, essential to the characterization of technological plasmas of interest in many fields, relies on the fundamental information about the collision integrals for the binary interactions in the system. The phenomenological approach has been demonstrated to provide a very useful theoretical framework for the derivation of transport cross sections, and in turn collision integrals, by a physics-sound description of the chemical species interaction. The features of the method and its validation are here briefly reviewed and the impact of the recent generalization of the correlation formulas on collision integrals for interactions involving Si species is estimated.

## 1. Introduction

Technologies based on low-temperature plasmas are gaining ground in a variety of fields [1] ranging from energy [2,3], environment [4], aerospace for entry conditions [5,6] and propulsion [7], material sciences [8], plasma medicine [9,10] and agriculture [11]. The very peculiar performances of plasmas in non-equilibrium conditions meet, in many cases, the requirements of sustainability, efficiency and selectiveness, mandatory for any future enabling technology. On the other hand, thermal plasmas are also of technological relevance, as plasma torches for waste disposal [12], plasma arcs for welding [13], laser-induced breakdown spectroscopy (LIBS) [14,15], plasma spraying [16], plasma cutting [17], circuit interruption [18,19] and mineral processing [20]. In all these applications, fluid dynamic modeling coupled with the chemistry, through advanced state-to-state or multi-temperature kinetic approaches [21], represents a valuable tool for the predictive simulation of the plasma properties and behavior, potentially assisting reactor design rather than material fabrication, novel-concept diagnostics or process control.

Characterization of the transport properties of a plasma is a fundamental requirement for estimating the transfer of momentum, energy, mass and charge and assessing specific physical interactions, such as the heat fluxes at the surface of a spacecraft entering the atmosphere [5]. Accurate values of viscosity, electric and thermal conductivity, with its components, and diffusion coefficients can be derived, in wide ranges of pressure/temperature conditions, in the framework of the Chapman–Enskog theory [22,23,24] where the microscopic collisional dynamics driving the transport is described by the collision integrals of binary interactions.

Collision integrals are thermal-averaged energy integrals of transport elastic cross sections and require the integration, through quantum, semiclassical or classical approaches, of the potentials describing, in the isotropic assumption, the interaction as a function of the inter-particle distance, i.e., the electronic states of the corresponding quasi-molecular system as the colliding partners approach. When open-shell species are involved, the number of states could be large, as predicted by the theory of electronic (angular and spin) momenta composition. The rigorous, *multi-potential* approach prescribes each allowed path of interaction properly accounted for, where the effective collision integral of (*ℓ*,*s*) order ($\sigma^{2}\Omega^{(\ell ,s)★}$) [22,23,24] results from each molecular state averaged by its statistical weight [24]. On the other hand, the interactions between molecular systems could also suffer of the isotropic assumption and it is worth mentioning a study [25,26,27,28,29,30,31,32] where attempts are made to derive accurate transport properties and rate coefficients of energy transfer processes by using molecular dynamic methods (QCT, quantum, …) on accurate potential energy surfaces for ground and excited states, this approach being limited to specific collisional pairs, whose electronic structure has been characterized with a high level of detail.

This explains why, despite the modern methods in theoretical chemistry, semi-empirical approaches are still widely used. This is the case of the *phenomenological approach* [33,34], based on the improved Lennard-Jones (ILJ) formulation combined with physics-sound correlation formulas for the potential parameter estimation, that allows an internally-consistent, analytical, full-range description of the intermolecular potential energy in the more and less stable configurations of the interacting system. Such a method is important since it suggests new experiments and ab initio calculations to test its validity and, eventually, to improve its formulation, and it can be applied also to predict the behavior of systems at increasing complexity, for which experimental and/or theoretical information is lacking. The phenomenological method has been validated for benchmark collision-pair interactions [35], demonstrating its applicability in the characterization of transport phenomena in planetary atmospheres entry conditions, representing environments with stable and unstable chemical species, as free radicals, open shell atoms, both in ground and excited electronic states, and ions [35,36,37,38,39]. It is worth mentioning that the approach can easily accomodate the physics of interactions involving species in electronic excited states, as demonstrated in Ref. [38] for the low-lying excited states of atomic nitrogen and oxygen and for the highly-excited atomic hydrogen pairs, H(*n*)-H(*n*). In fact, excited states, especially in non-equilibrium plasmas, could relevantly modify the transport properties of the mixture and this aspect has recently been an object of renewed interest [40,41,42].

Since its first publication, the phenomenological approach significantly impacted the literature and it has been exploited for the investigation of complex technological plasmas, ranging from high-voltage circuit breakers [43,44,45] to new functional material in processing applications [46,47], extreme ultraviolet lithography [48], and biomass renewable energy sources [49], up to the most recent implementations of the method for the construction of an extended database of neutral C, H, N,O, and Si-containing species to be included in the Gordon–McBride NASA-9 thermodynamic database [50] and for a comprehensive collision-specific Variable Soft Sphere (VSS) parameter database for accurate simulation of transport properties in DSMC (Direct Simulation Monte Carlo) [51].

In the present paper, in Section 2, the theoretical foundations of the phenomenological approach and the working equations are recalled, while in Section 3, the recent efforts in developing a generalized expressions for correlation formulas, in order to accurately describe the effective number of electrons actually participating in determining the polarization in atoms and molecular systems characterized by the presence of high-atomic-number elements, are presented. In Section 4, the accuracy of the phenomenological collision integrals in comparison with multi-potential results from the literature is briefly reviewed and, for interactions involving Si species, the sensitivity of collision integrals to the use of accurate values of $N_{\mathrm{eff}}$ is investigated, where $N_{\mathrm{eff}}$ is a parameter modulating the strength of the attraction since it defines the effective number of electrons undergoing polarization (see below). Conclusions are drawn in Section 5.

## 2. The Phenomenological Approach

The phenomenological approach is solidly based on molecular beam scattering experiments probing the features of single two-body collision events between gas phase atoms and molecules [33,34,52]. The analysis of the experimental findings, controlled by pure van der Waals (vdW) forces (for convenience defined as combination of size (exchange-Pauli) repulsion with dispersion attraction), suggested the representation of the equilibrium distance $R_{m}$ in the interacting complex, formed by two neutral partners, and of its binding energy ε by correlation formulas, given in terms of polarizability $\alpha_{a}$ and $\alpha_{b}$ of the partners (*a*,*b*) involved. Specifically,

(1) $$R_{m}=1.767\cdot \frac{\alpha_{a}^{\left(1/3\right)}+\alpha_{b}^{\left(1/3\right)}}{{\left(\alpha_{a}\cdot \alpha_{b}\right)}^{\gamma }}$$

where $R_{m}$ is in Å, α is in Å^3^ and γ = 0.095 for all systems. Such a relation exploits the basic concept [33] that the polarizability of each interacting partner simultaneously represents its volume (contributing to the size repulsion) and the probability of induced multipole formation (controlling the attraction). Accordingly, $R_{m}$ depends on the balance between repulsion, represented as sum of size contributions of the two partners, given as cube root of their polarizability, and attraction, proportional of the product of polarizabilities. Moreover,

(2) $$\varepsilon =0.72\frac{{\left(C_{6}\right)}_{\mathrm{eff}}}{R_{m}^{6}}$$

Values of the *effective-attractive* ${\left(C_{6}\right)}_{\mathrm{eff}}$ coefficient have been extracted [33,34] from the analysis of the average integral cross sections, measured in absolute scale for many systems with the same methodology. Such values provide the global dispersion attraction, $V_{\mathrm{disp}}\left(R\right)$, in the range of separation distance R mainly probed by the scattering experiments. Obtained values for ${\left(C_{6}\right)}_{\mathrm{eff}}$ depend on the balance of several long-range two-body attraction contributions (induced dipole–induced dipole, induced dipole–induced quadrupole, induced quadrupole–induced quadrupole, etc.) properly damped by the emergence of overlap effects [33]. The formula already adopted, which represents an extension of the semi-empirical Slater–Kirkwood equation (see Ref. [33] and references therein), is

(3) $${\left(C_{6}\right)}_{\mathrm{eff}}\left[\mathrm{meV}\cdot {Å}^{6}\right]=15700\cdot \frac{\alpha_{a}\cdot \alpha_{b}}{\sqrt{\frac{\alpha_{a}}{{\left(N_{\mathrm{eff}}\right)}_{a}}}+\sqrt{\frac{\alpha_{b}}{{\left(N_{\mathrm{eff}}\right)}_{b}}}}$$

which provides results consistent with the experimental determination [33]. Such an extension was basically provided by a general definition of the effective electron number ($N_{\mathrm{eff}}$) of each partner undergoing polarization during the interaction, that is participating to the induced multipole formation. It is worth noticing that the ratios under squared root at the denominator in the previous equation are dimensionally equal to ${\mathrm{length}}^{3}/\mathrm{number}\;\mathrm{of}\;\mathrm{electrons}$, i.e., the inverse of the electron density. This represents a basic concept, worth of further future investigation, to explain some peculiar behaviors observed in the interaction of very small ions, as the case of Be^2+^ and the limit case of H^+^, with neutral partners.

In the above equations, also known as scaling laws, numerical coefficients have been obtained on phenomenological ground [33].

It is of relevance to note that Equation (1) has been recently confirmed, for symmetric noble gas dimers, by a refined quantum mechanical treatment [53]. In particular, from such an equation, it emerges that $R_{m}$, equal to two times the vdW radius, depends on $\alpha^{1/7}$. This finding and the proportionality constant proposed several years ago are in perfect agreement with the theoretical results. Moreover, the ε value in Equation (2) corresponds to approximately 70% of the attraction in $R_{m}$ defined by ${\left(C_{6}\right)}_{\mathrm{eff}}$ and its reduced effect is ascribable to the contribution of size repulsion due to the emerging overlap effects. An extended meaning of such reduction, given in the generalization of correlation formulas (see below), also suggests how to evaluate the true induced dipole–induced dipole $C_{6}$ coefficient from the obtained ${\left(C_{6}\right)}_{\mathrm{eff}}$ value to be compared with data from the literature.

Previous formulas have been also extended to neutral-ion (N-I) systems [34,35], introducing the parameter ρ, representative of the relative role of dispersion and induction (ion–induced dipole) attraction components in proximity to the equilibrium distance. In particular, the following relations (see Refs. [34,35] and references therein) were proposed:

(4) $$\rho =\frac{\alpha_{I}}{z^{2}\cdot \left[1+{\left(\frac{2\alpha_{I}}{\alpha_{N}}\right)}^{2/3}\right]\cdot \alpha_{N}^{1/2}}$$

(5) $$R_{m}=1.767\cdot \frac{\alpha_{I}^{\left(1/3\right)}+\alpha_{N}^{\left(1/3\right)}}{{\left(\alpha_{I}\cdot \alpha_{N}\cdot \left[1+\frac{1}{\rho }\right]\right)}^{\gamma }}$$

(6) $$\varepsilon \left[\mathrm{meV}\right]=0.72\frac{{\left(C_{4}\right)}_{\mathrm{eff}}}{R_{m}^{4}}$$

where *z* is the ion charge and ${\left(C_{4}\right)}_{\mathrm{eff}}$ is an effective ion–induced dipole attraction coefficient, namely including also the dispersion contribution, defined as

(7) $${\left(C_{4}\right)}_{\mathrm{eff}}\left[\mathrm{meV}\;{Å}^{4}\right]=7200\cdot z^{2}\cdot \left(1+\rho \right)\cdot \alpha_{N}$$

The extension to ion–ion cases, where the leading long-range attraction is the Coulomb component, has been also attempted (see Refs. [34,54] and references therein).

### 2.1. Improvement of the Phenomenological Method

The improvement of the phenomenological method consists in the search of possible empirical and semi-empirical relations, of general validity, between leading potential components—in particular, the parameters involved in their formulation—and some fundamental physical properties of the interacting partners. Such relations are useful to better define on a more general ground, strength, range and anisotropy the non-covalent intermolecular interaction, whose leading components have a “physical” nature, with particular attention addressed to their radial and angular dependence. As in the past [33,34,35], our strategy is to provide formulas defined in terms of few quantities having a proper physical meaning, whose results show a transferability character. Note that in some cases, the basic features of the interaction can substantially differ from the predictions of the phenomenological method and, usually, the deviation provides information on the strength of additional (“chemical”) contributions to the non-covalent interaction [34].

### 2.2. Basic Features of the ILJ Formulation

In the last years, the improved Lennard-Jones (ILJ) function has been extensively used to describe, in systems at increasing complexity, the non-covalent (supramolecular) long range part of the intermolecular interaction potential in a wide range of separation distances and relative orientations of involved neutral and ionic partners [54,55,56,57]. Note that vdW represents the simplest case of non-covalent interaction between two neutral partners. The comparison between predicted and theoretical/experimental features of the formed weak intermolecular bond has been important to test and improve ILJ. The improvement consisted in a better characterization of the variability range of the adopted potential formulation. Accordingly, the last version of ILJ [54], given in the reduced form (that is scaling the interaction energy *V* for the potential well depth value ε and the separation distance *R* for the equilibrium value $R_{m}$), plays the form

(8) $$\frac{V_{\mathrm{ILJ}}}{\varepsilon }=\frac{m}{n(x)-m}{\left(\frac{1}{x}\right)}^{n(x)}-\frac{n(x)}{n(x)-m}{\left(\frac{1}{x}\right)}^{m}$$

where $x=R/R_{m}$, and *m* is equal to 6, 4 and 1 for neutral–neutral, ion–neutral and ion–ion systems respectively [54]. The systems of interest are mostly those binding through the balance of size repulsion, dominant at short range, namely for *x* lower than 1, with dispersion–induction attractions and electrostatic effects (due to charges and permanent multipole interactions) that are prevailing for reduced distance *x* values much larger than 1. For neutral–neutral systems, for which *m* = 6, ILJ provides asymptotically the value of the dipole–dipole $C_{6}$ dispersion coefficient, defined as

(9) $$C_{6}=\varepsilon \cdot R_{m}^{6}$$

For ion–neutral systems, for which *m* = 4, the leading ion–induced dipole attraction $C_{4}$ coefficient plays the form

(10) $$C_{4}=\varepsilon \cdot R_{m}^{4}$$

For ion–ion systems, for which *m* = 1, according to the Coulomb law, the leading attraction $C_{1}$ coefficient is formulated as

(11) $$C_{1}=\varepsilon \cdot R_{m}$$

The ILJ function has been also extended to include the anisotropy of the interaction. The extension has been performed in atom–molecule and molecule–molecule systems by adopting Legendre polynomials and spherical harmonic expansions for $R_{m}$, ε and β quantities [31,55]. Moreover, the n(x) term can be defined on a more general way as

(12) $$n\left(x\right)=ax^{2}+\beta$$

For neutral–neutral partners, the classical Lennard-Jones, LJ (12,6) model is effectively re-obtained if the *a* factor (whose value is 4 in the original form of ILJ [34,35,54]) is assumed to be zero and the β term is fixed to the value of 12.0.

It is possible also to verify that using *a* = 4.0, β = 8 and *m* = 6 at the equilibrium distance, namely at *x* = 1, first and second derivatives of ILJ coincide with those of LJ (in particular, the reduced force constant amounts to 72 (typical of a LJ (12,6)). However, it should be noted that, although in some cases, at the equilibrium distance, both ILJ and LJ have the same force and force constant, asymptotically, LJ provides a long-range $C_{6}$ coefficient a factor 2 larger than the correct value, while that predicted by the behavior of ILJ, with *a* = 4.0, is correct [54]. Also, the short-range repulsion is overestimated by IL (12,6). Similar considerations apply to systems involving positive and negative ions.

Finally, the separation distance σ, where the interaction potential becomes zero for the balance of attraction with repulsion, is a basic parameter usually adopted to represent the collision diameter and related gas-kinetic cross section. However, σ must be related to $R_{m}$ with a proportionality constant depending on the nature of the interaction partners. ILJ provides the following relations for the various systems: neutral–neutral, σ = 0.890 (±0.011)$\cdot R_{m}$; ion–neutral, 0.860 (±0.012)$\cdot R_{m}$; ion–ion, 0.765 (±0.013)$\cdot R_{m}$, where the ±quantity depends on the modulation of the potential well shape by the β parameter. Such relations represent an extension of those previously reported in Ref. [58].

## 3. Generalization of the Correlation Formulas

The generalization of the correlation formulas, providing range and strength of the non-covalent (supramolecular) interaction in neutral–neutral systems at increasing complexity, must start from a more extended-complete definition of the effective number of electrons ($N_{\mathrm{eff}}$) participating to the atomic or molecular polarization (also defined as induced multipole formation) in presence of the other partner. In particular, $N_{\mathrm{eff}}$ appears in Equation (3), adopted [33] to describe the dispersion coefficient, ${\left(C_{6}\right)}_{\mathrm{eff}}$, in terms of polarizability $\alpha_{i}$ of interacting neutral partners (*i* = *a*, *b*). Note also that the true induced dipole–induced dipole $C_{6}$ dispersion coefficient is expected to be in the range of 72–80% of ${\left(C_{6}\right)}_{\mathrm{eff}}$. The numerical reduction factor is dependent on the combined effect of size repulsion role with the damping of higher order dispersion coefficients to be subtracted from the ${\left(C_{6}\right)}_{\mathrm{eff}}$ value. Such combined effect shows a limited dependence on the investigated system. In particular, we found that 0.72 is a reduction factor (Equation (2)) covering an ample phenomenology [33,34,35,54]. Here, in the variability range 0.72–0.80, we include possible inaccuracies of our approach, the global uncertainty of the polarizabilities $\alpha_{i}$ and also of data from literature used as references [59,60,61,62,63].

### 3.1. Extension of the $N_{\mathrm{eff}}$ Definition

Previously [33], the attention focused on the behavior of atoms up to Xe and of light molecules. For a proper generalization of $N_{\mathrm{eff}}$’s definition, it is useful to distinguish the cases of atoms from those of molecules.

#### 3.1.1. Atoms

From the equation reported below, which is the same provided in Ref. [33], it appears that $N_{\mathrm{eff}}$ depends on a proper combination of external, $N_{\mathrm{ext}}$, and internal, $N_{\mathrm{int}}$, electrons numbers

(13) $$\frac{N_{\mathrm{eff}}}{N_{\mathrm{ext}}}=1+\left(1-\frac{N_{\mathrm{ext}}}{N_{\mathrm{int}}}\right)\cdot {\left(\frac{N_{\mathrm{int}}}{N_{\mathrm{tot}}}\right)}^{2}$$

where $N_{\mathrm{tot}}$ = $N_{\mathrm{ext}}$ + $N_{\mathrm{int}}$ = atomic number (A.N.)

For the simplest cases of H and He, $N_{\mathrm{eff}}$ amounts to 1 and 2, respectively, and for elements of the second and third period, the results coincide with those plotted in Figure 1 of Ref. [33]. Moreover, for elements of the other periods, formed also by *d* and *f* electrons, some additional features of involved electrons must be stressed to obtain a further generalization of the $N_{\mathrm{eff}}$ formulation. Particular attention must be addressed to atoms with outer electrons exhibiting similar energy, although they are described by a different combination of quantum numbers. The following cases are here emphasized:

a The criteria provided in Ref. [33], based on closeness and relative energy of ns and (n−1)d orbitals, suggest that for the first half of the first and second periods of transition elements, the weighted sum of *N* electrons occupying outer *s* and *d* orbitals, having comparable energy, must be adopted in the definition of $N_{\mathrm{ext}}$, namely $N_{\mathrm{ext}}$ = $N_{ns}$+0.60 $N_{(n-1)d}$. For all elements of the second half, A.N. increases, *d* electrons assume a more internal character with respect to the *s* electrons, and therefore, $N_{\mathrm{ext}}$ tends to play the constant value of 5. Further increasing A.N., the $N_{\mathrm{ext}}$ evaluation involves only the population of ns and np, since (n−1)d become fully internal orbitals. For instance, for Zn (A.N. = 30, [Ar] $3d^{10}4s^{2}$), $N_{\mathrm{ext}}$ = 5, $N_{\mathrm{int}}$ = 25, $N_{\mathrm{eff}}$ = 7.8; for Ga (A.N. = 31, [Ar]$3d^{10}4s^{2}4p^{1}$), $N_{\mathrm{ext}}$=3, $N_{\mathrm{int}}$ = 28, $N_{\mathrm{eff}}$ = 5.2; for Br (A.N. = 35, [Ar]$4d^{10}5s^{2}5p^{5}$), $N_{\mathrm{ext}}$ = 7, $N_{\mathrm{int}}$ = 28, $N_{\mathrm{eff}}$ = 10.4 (see also Figure 1 in Ref. [33]) and for I (A.N. = 53, [Kr]$4d^{10}5s^{2}5p^{5}$), $N_{\mathrm{ext}}$ = 7, $N_{\mathrm{int}}$ = 46, $N_{\mathrm{eff}}$ = 11.5.
b Similar considerations apply to heavier elements including *d* and *f* electrons, leading to $N_{\mathrm{ext}}$ = $N_{ns}$+$N_{np}$+0.60 $N_{\left[(n-1)d,(n-2)f\right]}$ . The third term contributes up to 6. Note that all other electrons, including those populating (n−2)d orbitals, are considered internal. For instance, for Tm (A.N. = 69, [Xe]$4f^{13}5d^{0}6s^{2}$), $N_{\mathrm{ext}}$ = 8, $N_{\mathrm{int}}$ = 61, $N_{\mathrm{eff}}$ = 13.4; for Hg (A.N. = 80, [Xe]$4f^{14}5d^{10}6s^{2}$), $N_{\mathrm{ext}}$ = 8, $N_{\mathrm{int}}$ = 72, $N_{\mathrm{eff}}$ = 13.8 (15.5, value from literature [63]); For Rn (A.N. 86, [Xe]4f^14^5d^10^6s^2^6p^6^), $N_{\mathrm{ext}}$ = 14, $N_{\mathrm{int}}$ = 72 , $N_{\mathrm{eff}}$ = 21.9 (19.0, value from literature [59]).
c As for case b), for elements heavier than Rn, including *d* and *f* electrons, $N_{\mathrm{ext}}$ = $N_{ns}$+$N_{np}$+0.60 $N_{\left[(n-1)d,(n-2)f\right]}$. The third term always contributes up to 6.0. For instance, for U (A.N. = 92, [Rn]$5f^{3}6d^{1}7s^{2}$), $N_{\mathrm{ext}}$=4.4, $N_{\mathrm{int}}$ = 87.6, $N_{\mathrm{eff}}$ = 8.2; for Og (A.N. 118, [Rn]5f^14^6d^10^7s^2^7p^6^), $N_{\mathrm{ext}}$ = 14, $N_{\mathrm{int}}$ = 104, $N_{\mathrm{eff}}$=23.4 (24.0, value from literature [60]).

#### 3.1.2. Molecules

For molecules, it is also convenient to distinguish two different cases:

a For molecules formed by elements of first and second periods, the previously provided formula [33] works correctly. It is defined as
  (14) $$\frac{N_{\mathrm{eff}}}{N_{t}}=1-\frac{N_{b}\cdot N_{\mathrm{nb}}}{N_{t}^{2}}$$
  where $N_{t}$ is the sum of bonding, $N_{b}$, and non-bonding, $N_{\mathrm{nb}}$, electrons. For the simplest H_2_ case: $N_{b}$ = 2, $N_{\mathrm{nb}}$ = 0, $N_{t}$ = 2 and $N_{\mathrm{eff}}$ = 2. Such formula does not include any contribution of internal electrons, which is small for lighter molecules, but it becomes relevant for heavier molecules.
b The generalization of this approach, covering a more ample phenomenology, leads to the following relation:
  (15) $$\frac{N_{\mathrm{eff}}}{N_{t}}=1-\frac{N_{b}\cdot N_{\mathrm{nb}}^{★}}{N_{t}^{2}}$$
  where $N_{t}$ is the sum of bonding, $N_{b}$, and non-bonding, $N_{\mathrm{nb}}^{★}$, electrons. Here, $N_{\mathrm{nb}}^{★}$ arises from the combination of external $N_{\mathrm{nbe}}$ and internal $N_{\mathrm{nbi}}$, non-bonding contributions. In particular, $N_{\mathrm{nb}}^{★}$ can be evaluated from the following equation:
  (16) $$\frac{N_{\mathrm{nb}}^{★}}{N_{\mathrm{nbe}}}=1+\left[1-\frac{N_{\mathrm{nbe}}}{N_{\mathrm{nbt}}}\right]\cdot {\left[\frac{N_{\mathrm{nbi}}}{N_{\mathrm{nbt}}}\right]}^{2}$$
  where $N_{\mathrm{nbt}}=N_{\mathrm{nbe}}+N_{\mathrm{nbi}}$.
  The proposed generalization is under validation through a thorough comparison of the phenomenological long-range dispersion attraction coefficients $C_{6}$ with data in the literature, considering collision pairs involving molecular partners of variable complexity. However, with respect to the previous evaluation, for light diatomic molecules as N_2_ and O_2_, $N_{\mathrm{eff}}$ increases from 7.60 to 7.93 and from 9.33 to 9.60, respectively. More pronounced increases are expected for molecules involving a large number of internal non-bonding electrons.

## 4. Collision Integrals

The estimation of the coefficients describing the transport of momentum, mass, heat and charge in plasmas relies on the elementary binary interactions entering into the collision integrals in the Chapman–Enskog theory. Classical collision integrals, $\Omega^{\left(\ell ,s\right)}$, entering the working equations, result from a threefold integration: on the inter-particle distance *r*, on the impact parameter *b* and on the reduced energy E/kT [22,24].

The phenomenological approach provides the theoretical framework for the straightforward derivation of a consistent and accurate collision integral database for neutral–neutral and neutral–ion interactions for virtually any collision-pair. For ion–parent-neutral collision pairs, e.g., O^+^–O or N^+^–N, the contribution to the odd-order collision integrals from the resonant charge-exchange process must be also accounted for [24]. It is worth mentioning that, in plasmas, a role is played also by electron–neutral and charged–charged interactions, governing the transport in the high-temperature region, above the onset of ionization equilibria; thus, a general approach to transport coefficients must encompass the estimation of collision integrals for these two classes of interactions [24]. The electron–neutral pairs need a quantum characterization, and momenta of the elastic transport cross sections are usually derived from differential electron elastic scattering cross sections from experiments or theoretical quantum approaches, while charged–charged interactions are described using the screened-Coulomb (Yukawa) potential, for which collision integrals, parametrically depending on the Debye length, were calculated for repulsive and attractive interactions [64,65] and fitted with analytical expressions [24,66].

For the phenomenological approach, reduced collision integrals up to high orders, namely (ℓ,s) = (4,4), have been calculated in a wide range of reduced temperatures considering integer values of β in its range of variability for both neutral–ion and neutral–neutral interactions and fitted with a suitable bi-dimensional function, so that collision integrals can be straightforwardly obtained once the tuple of parameters is known (ε, $R_{m}$, *m*, β) [35,36].

**Figure 1:**
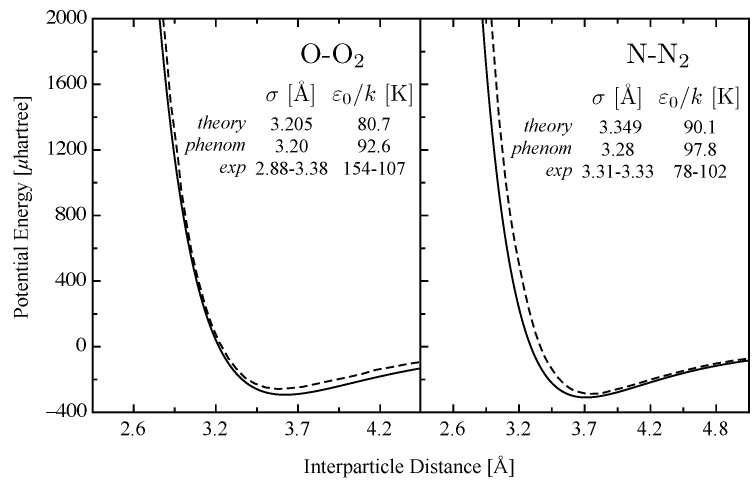
Interaction potentials for O–O_2_and N–N_2_ colliding systems. Solid lines represent phenomenological approach, dashed lines represent accurate *effective* isotropic potential from Ref. [69]. Potential parameters from theory [69], phenomenological approach and experiments [70,71]. Reprinted by permission from Springer Nature: (D’Angola et al. 2012) [68].

The validation of the method went through the estimation of benchmark collision-pair interactions relevant to the Earth atmosphere [35,36,67]. The phenomenological approach demonstrated its reliability and the comparison with recommended collision integrals was always satisfactory. An example is represented by O–O_2_ and N–N_2_ interactions. These two systems are of interest for dissociation regimes and govern the properties at temperatures relevant to the onset of dissociation equilibria [68]. In Figure 1, the ILJ potentials for the two colliding pairs are compared with the effective isotropic interaction potentials, derived from ab initio calculations at MRCI level accounting for the dependence on the angle of approach [69], showing a strikingly good agreement.

**Figure 2:**
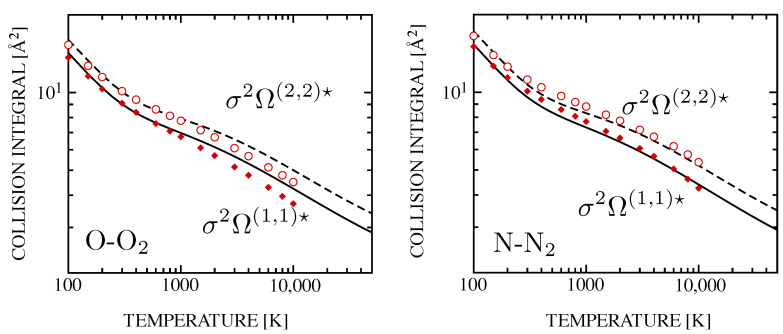
Phenomenological diffusion (solid line) and viscosity-type (dashed) collision integrals for O–O_2_ (**left**) and N–N_2_ (**right**) colliding systems, compared with accurate results, $\sigma^{2}\Omega^{(1,1)★}$ (close diamonds) and $\sigma^{2}\Omega^{(2,2)★}$ (open circles), from the literature [69]. (*y*-axis goes from 2 to 20 Å^2^). Reprinted by permission from Springer Nature: (Pirani et al. 2019) [67].

The accuracy of the phenomenological potential reflects on the collision integrals, presented in Figure 2, that reproduce the recommended results by [69] in a wide range of temperatures. In particular, the low-temperature region, dominated by the long-range attractive part of the interaction, tells about the predictive character of the correlation formulas in the estimation of the potential parameters, well comparing with the values derived by the accurate procedure and also with experimental results [70,71], as appreciable in Figure 1.

**Figure 3:**
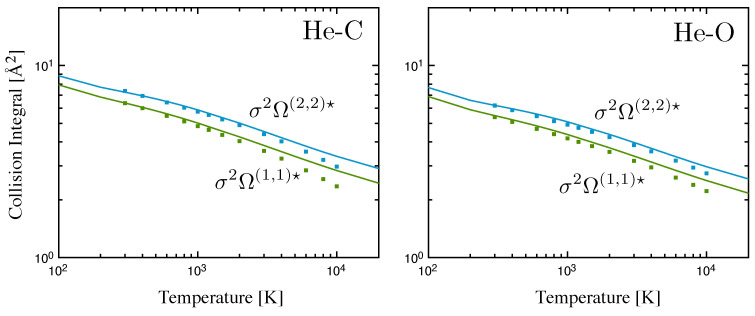
Diffusion- (green) and viscosity-type (blue) collision integrals for helium–carbon and helium–oxygen interactions. Solid lines represent phenomenological method, markers represent multi-potential approach from Ref. [72]. Reprinted by permission from Springer Nature: (Pirani et al. 2023) [56] under Creative Commons CC BY license.

Another example of the good performances of the method is relevant to the interactions of helium with ground state C and O [56]. The phenomenological collision integrals for He–C and He–O collision pairs are displayed in Figure 3 and compared with the accurate results obtained in Ref. [72] with a traditional multi-potential approach.

The phenomenological approach also allowed the derivation of transport properties of plasmas including silicon species [50,73,74]. In fact, silica and silicon carbide (SiO_2_, SiC) are chemical components of the tiles in ablative thermal protection systems of space vehicles subject to thermal shock during the (re)entry in planetary atmospheres; therefore, the accurate knowledge of transport coefficients in wide temperature ranges is relevant to the high-fidelity fluid-dynamic simulation of the heat load at the surface, providing a virtual laboratory for material testing and TPS design. The characterization of silicon-containing plasmas could also find application in the investigation of chondritic meteorites undergoing ablation when entering the Earth atmosphere at high velocities [74].

In Table 1, the polarizability and $N_{\mathrm{eff}}$ values predicted by Equation (15) for silicon-based atomic and molecular species are presented. For polarizability, references in the literature are reported; in the case of SiC, the value has been estimated through an empirical formula, accounting for effective polarizability of atomic components in the molecule [75]. In the last column of Table 1, the old values used in Refs. [73,74] are also reported. The *old* estimations markedly overestimate $N_{\mathrm{eff}}$ for silicon dimer and trimer, while for SiC, the value is slightly different from the accurate one.

**Figure 4:**
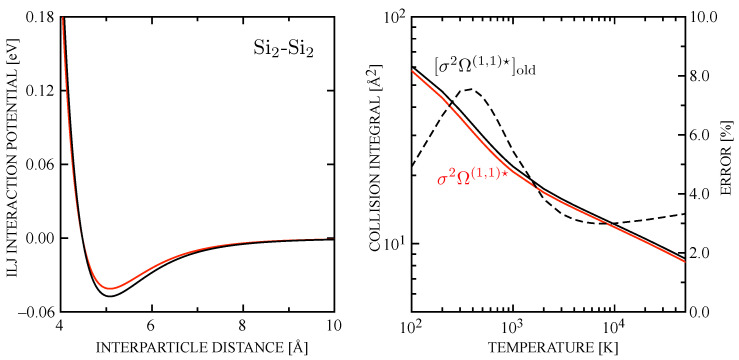
(**left**) ILJ potential for Si_2_-Si_2_ collision-pair obtained with the $N_{\mathrm{eff}}$ by Equation (15) (red curve) compared with the potential with the value estimated by Equation (14) (black curve); (**right**) $\sigma^{2}\Omega_{{\mathrm{Si}}_{2}-{\mathrm{Si}}_{2}}^{(1,1)★}$ collision integrals as derived in Ref. [74] (black curve) compared with results calculated with the correct value of $N_{\mathrm{eff}}$ by Equation (15) (red curve) (the percentage error, with reference to the right axis, is also represented (dashed curve)).

In Figure 4, the Si_2_-Si_2_ collision pair is considered. The ILJ potential obtained with the $N_{\mathrm{eff}}$ by Equation (15) (Figure 4 left panel), when compared with the old estimation, exhibits a reduction in the well depth ε (relevant potential parameters are presented in Table 2). The diffusion-type collision integrals (Figure 4 right panel) are accordingly lower with respect to the $\sigma^{2}\Omega_{{\mathrm{Si}}_{2}-{\mathrm{Si}}_{2}}^{(1,1)★}$ derived in Ref. [74], due to the lower long-range attractive character of the interaction governing the low-temperature profile of the collision integral. The error affecting the old estimation, also displayed in the figure, remains below 8% peaking at 400 K and decreasing to about 3% at higher temperatures, where the short-range repulsive interaction dominates.

**Figure 5:**
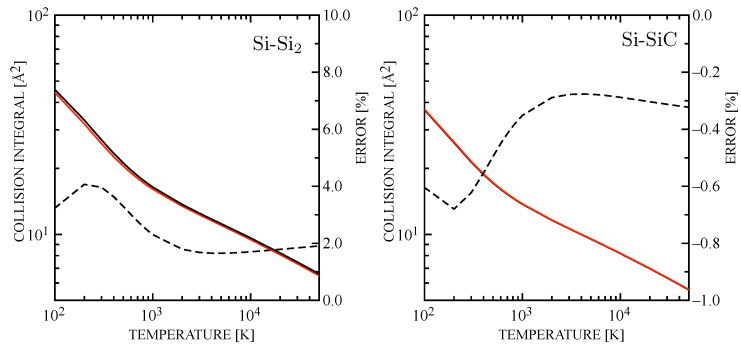
Diffusion-type collision integrals for Si-Si_2_ (**left**) and Si-SiC (**right**) interactions. The percentage error is also represented as in Figure 4. The curve symbols are consistent with Figure 4.

**Figure 6:**
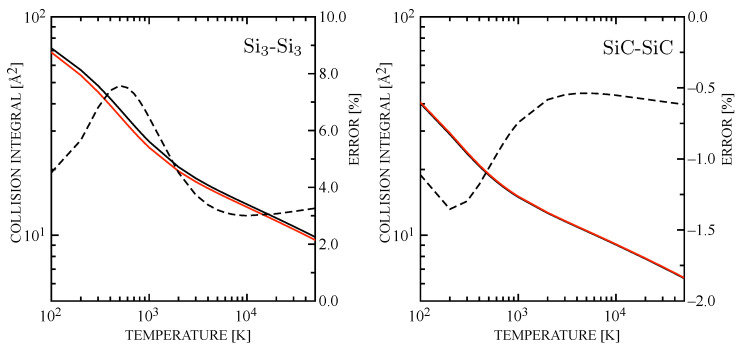
Diffusion-type collision integrals for Si_3_-Si_3_ (**left**) and SiC-SiC (**right**) interactions. The percentage error is also represented as in Figure 4. The curve symbols are consistent with Figure 4.

Figure 5 and Figure 6 illustrate a systematic comparison of the $\sigma^{2}\Omega^{(1,1)★}$ for Si–molecules and molecule–molecule collision pairs. The Si_3_-Si_3_ interaction is rather similar to the dimer case, while Si-Si_2_ shows that the error is significantly compressed and it becomes very small (and opposite in sign) for the interactions involving SiC. Therefore, it can be concluded that the incorrect use of Equation (14) in Refs. [73,74] does not invalidate the analysis on the transport properties that are expected to be slightly modified by the use of newly estimated collision integrals.

## 5. Conclusions

The high-fidelity fluid dynamic simulation of space vehicle entry conditions in planetary atmosphere requires accurate values of the transport properties of the plasma in the shock, characterized by the presence of many chemical species formed by reactive processes and also ablated from the materials of the TPS. The high chemical complexity of the resulting system poses difficulty in the derivation of consistent and complete collision integral databases and different strategies have been proposed in the past. The phenomenological approach is nowadays widely used in the community, due to its predictive character and the possibility to rely on correlation formulas based on the physical properties of colliding partners for the description of the interaction potential also when exotic chemical species are involved. In the present paper, the phenomenological method and its performances in reproducing accurate multi-potential results for benchmark systems are reviewed. Furthermore, a recent generalization of the correlation formulas, allowing the safe application of the method to chemical species containing heavy elements, is presented and the influence of the generalized $N_{\mathrm{eff}}$ definition on collision integrals for silicon species is critically assessed, confirming that the error in collision integrals, derived in the past with the straight use of correlation formulas strictly valid only for chemical species containing elements of the I and II period, is small.

## Figures and Tables

**Table 1:** Polarizability and $N_{\mathrm{eff}}$ value predicted by Equation (15) for chemical species relevant to silicon systems. For molecules also, the value ${\left[N_{\mathrm{eff}}\right]}_{\mathrm{old}}$, from previous estimates, is reported.

**Atom**	$N_{\mathrm{tot}}$	α [Å^3^]	$N_{\mathrm{ext}}$	$N_{\mathrm{int}}$	$N_{\mathrm{eff}}$			
Si	14	5.38 [76]	4	10	5.22			
**Molecule**	$N_{\mathrm{tot}}$	α [Å^3^]	$N_{b}$	$N_{\mathrm{nbe}}$	$N_{\mathrm{nbi}}$	$N_{\mathrm{nb}}^{★}$	$N_{\mathrm{eff}}$	${\left[N_{\mathrm{eff}}\right]}_{\mathrm{old}}$
Si_2_	28	12.58 [77]	4	4	20	6.31	7.86	10.44
Si_3_	42	15.66 [77]	6	6	30	9.47	11.80	15.66
SiC	20	6.63 [75]	4	4	12	5.69	7.34	6.96

**Table 2:** ILJ potential parameters for silicon species interactions obtained with the correct $N_{\mathrm{eff}}$ values predicted by Equation (15) and with values estimated by Equation (14) in Refs. [73,74].

Interaction	ε [eV]	$R_{m}$	β	${\left[\varepsilon \right]}_{\mathrm{old}}$ [eV]
Si_2_-Si_2_	0.0411	5.080	7.075	0.0474
Si-Si_2_	0.0264	4.828	6.659	0.0286
Si-SiC	0.0226	4.568	6.701	0.0223
Si_3_-Si_3_	0.0580	5.242	6.999	0.0668
SiC-SiC	0.0264	4.635	7.331	0.0257

## Data Availability

The original contributions presented in this study are included in the article. Further inquiries can be directed to the corresponding authors.
